# Modern Technologies in the Rehabilitation of Patients with Multiple Sclerosis and Their Potential Application in Times of COVID-19

**DOI:** 10.3390/medicina57060549

**Published:** 2021-05-30

**Authors:** Ewa Zasadzka, Tomasz Trzmiel, Anna Pieczyńska, Katarzyna Hojan

**Affiliations:** 1 Department of Occupational Therapy, Poznan University of Medical Sciences, Swiecickiego Street 6, 60-781 Poznan, Poland; ezasad@ump.edu.pl (E.Z.); ttrzmiel@ump.edu.pl (T.T.); apieczynska@ump.edu.pl (A.P.); 2 Department of Rehabilitation, Greater Poland Cancer Centre, 15 Garbary Street, 61-866 Poznan, Poland

**Keywords:** multiple sclerosis, telerehabilitation, virtual reality, tele-exercises, physiotherapy, occupational therapy, SARS-CoV2

## Abstract

*Background and Objectives*: The COVID-19 pandemic required the adoption of new technologies to improve access to healthcare at an unprecedented speed, as social distancing became mandatory. The aim of this systematic review was to analyze the effectiveness of using new technologies in the rehabilitation of multiple sclerosis (MS) patients and discuss their potential role during the COVID-19 pandemic. *Material and Methods*: The studies were identified by searching two online databases—PUBMED and Web of Science. Combinations of the key words “Multiple sclerosis” and “e-health”; “Multiple sclerosis” and “virtual reality”; “Multiple sclerosis” and “telerehabilitation”; “Multiple sclerosis” and “new technologies”; “Multiple sclerosis” and “tele-exercise” were used to find suitable publications. *Results*: A total of 17 studies were included. Although the overall number of participants in all the studies was 904, two of the studies were conducted on the same group. Thus, a total of 854 participants were involved in the studies included. All participants were diagnosed with MS. In 10 studies, participants had to be diagnosed according to the McDonald criteria. Of the included studies: five involved intervention at participants’ home, six were conducted using Xbox Kinect, and seven studies reported no adverse outcomes. *Conclusion*: The review proves telerehabilitation to be an effective motivational tool to restore and maintain both physical and cognitive function in patients with MS. Remote communication technologies seem to be measures of high effectiveness in rehabilitating and supporting MS patients especially during the COVID-19 pandemic, as the traditional rehabilitation option is less accessible or in some cases inaccessible for these patients.

## 1. Introduction

Multiple sclerosis (MS) is a chronic inflammatory demyelinating disease of the central nervous system of unknown etiology and multifactorial origin [1]. MS is the most common chronic neurological disease among young adults in Europe and North America. Typical symptoms include fatigue, visual disturbances, balance and coordination problems, sensitivity disorders, spasticity, cognitive function and emotional disturbances, speech disorders, bladder and bowel problems, and sexual dysfunction [2,3]. Balance disorders and postural control impairment are among the most common motor disorders associated with MS, occurring in 20% of patients at the onset of disease and in 80% of patients with chronic MS symptoms [4]. Specifically, decreased speed and efficiency for timed walking is associated with occupational changes, loss of independence in activities of daily living (ADLs) and self-reported disability [5,6].

Neurorehabilitation programs are the most common therapies used to reduce disability and social disadvantage resulting from MS. Physical rehabilitation is one of the non-pharmaceutical interventions to improve walking ability, and to improve or maintain function, muscle strength and aerobic capacity. Additionally, higher levels of physical activity are also associated with lower mortality in people with MS [7,8,9]. Many neurological conditions are treated on an outpatient basis in hospitals, but this treatment is insufficiently effective because rehabilitation is time-limited [10]. The rehabilitation program for MS requires multiple sessions conducted over weeks or months. For people with motor disabilities and limited mobility, traveling to health centers for treatment can be difficult and often expensive, which can be an obstacle in accessing rehabilitation treatment. Due to the recent COVID-19 pandemic restrictions, access to inpatient treatment is limited and sometimes even impossible. The viral infection by SARS-CoV-2 has spread all over the world and the World Health Organization (WHO) declared an international public health emergency. To quickly diagnose and control such a highly contagious disease, it was recommended to isolate the infected individuals and develop diagnostic and therapeutic procedures based on epidemiological and clinical data of the patients. However, due to the worldwide spread of the virus, COVID-19 has become a serious problem in the medical community. The measures taken to prevent the spread of the virus such as social isolation, especially for the most vulnerable elderly and chronically ill people, have social and health consequences.

Unlike in the general population, the course of COVID-19 in MS patients is modified by several factors. For example, this chronic disease is often associated with several other health complications and chronic immunomodulatory treatment associated with immunosuppressive effects at various levels of the immune system and the cytokine network. In milder COVID-19 cases, the exacerbation of MS may be only temporary but the risk of relapse or progression of the disease is not negligible. This is the reason many countries developed risk groups (including MS patients) and emphasize the need for hygiene and social distancing [11]. In response to this situation, there has been a growing interest in developing e-health projects. In the context of e-health, telerehabilitation (TR) is the provision of rehabilitation services through electronic systems using information and communication technologies [12]. TR extends rehabilitation care beyond the hospital setting into a patient-friendly environment, helping to detect new limitations and evaluate the effectiveness of interventions in relation to daily life activities [12].

In recent years, technological innovations, such as PC-based and virtual reality (VR), have proven effective in improving motor and cognitive impairment in neurological patients, including those with MS [13,14]. VR increasing sensory feedback enhances specific cognitive domains, including attention, problem solving, working memory, praxia, and speed of information processing [15]. It has been shown that VR could improve neuropsychological deficits by stimulating and promoting brain plasticity, with a positive effect also on the motor components of MS subjects [16]. The major features of this multimedia technology are aimed at enabling interaction and sensory feedback in patients via a highly motivating multidimensional virtual environment in which the patient performs virtual daily activities or tasks. Patients rank the intensity and difficulty of these tasks by providing real-time information with regard to the objectives achieved [16].

Interactive multimedia technologies seem to be superior to traditional rehabilitation methods which are less available for patients due to limited access to hospitals. Recently, the number of studies concerning the use of VR and gamification consoles in neurorehabilitation has increased. Interactive multimedia technologies offer some advantages over traditional rehabilitation treatments either due to accessibility issues, geography, or treatment availability, providing motivational activities, therapeutic adherence, and treatment compliance [16].

In the face of the world-wide COVID-19 pandemic, it is vital to look for solutions that can ensure the continuity of treatment for patients with MS. One such therapeutic tool may be new technologies (such as VR and TR), which can be delivered safely at home, decreasing the risk of patient infection.

This systematic review aims to analyze the effectiveness of using new technologies in the rehabilitation of MS patients and to discuss their potential role in the era of the COVID-19 pandemic.

## 2. Material and Methods

A review of the literature was conducted in accordance with the Preferred Reporting Items for Systematic Reviews and Meta-Analyses (PRISMA) guidelines [17]. The procedures (search strategy, inclusion/exclusion criteria, and data extraction) were established and included in the protocol. An approval of an Ethics Committee is not required in such studies. The study was registered in the Research Registry and received Review Registry UIN: reviewregistry1138.

### 2.1. Search Strategy

The studies were identified by searching two online databases—PUBMED and Web of Science. The following combinations of the key words with Boolean operator “AND”: Multiple sclerosis AND e-health; Multiple sclerosis AND virtual reality; Multiple sclerosis AND telerehabilitation; Multiple sclerosis AND new technologies; Multiple sclerosis AND tele-exercise were used to find suitable publications. Two of the authors (TT and EZ) conducted their independent searches of the literature published in recent 10 years (between 1 January 2011 and 18 January 2021). Studies conducted on human subjects were identified, and the language was limited to English. Abstracts or unpublished reports were not considered. 

### 2.2. Inclusion and Exclusion Criteria

The inclusion criteria for the reports were as follows: (a) published in English in a journal with a review process, after 2010; (b) original research study with a control group or presentation of comparative pre- and post-therapy results of therapy involving new technology in MS patients; (c) clearly defined inclusion and exclusion criteria for the study groups and controls.

The following articles were excluded: (a) studies on populations including other patients than those with MS; (b) animal studies; (c) studies examining the effect of robotic intervention in MS patients; (d) studies lacking approval of local ethics committee; (e) studies with incomplete outcome data; (f) studies using additional therapies only in the study group, but not in the control group; (g) studies of undetermined type; (h) pilot studies and conference proceedings.

### 2.3. Quality Assessment

To determine methodological quality of the studies included, the Quality Assessment Tool for Quantitative Studies (QATQS) [18] was used. QATQS assesses eight domains of methodological quality: selection bias, study design, confounders, blinding, data collection methods, withdrawals and dropouts, intervention integrity, and analysis. The first six domains may be classified as ‘weak’, ‘moderate’ or ‘strong’, according to a reviewer’s dictionary. If one domain is rated as ‘weak’, the entire study is deemed ‘moderate’, if more than one section is ‘weak’, the study is considered automatically ‘weak’, and if neither section is ‘weak’, the study is rated as ‘strong’. The intervention integrity section helps to answer the question of the risk of overestimating or underestimating the intervention. This may be a consequence of e.g., delivering interventions to different participants in a heterogeneous manner, or accidental receipt of an intervention by a person from the control group.

The assessments were performed independently by two authors (TT and EZ). If agreement on the quality assessment could not be reached by the two authors, the third author was consulted (KH).

### 2.4. Data Extraction

The following data were extracted from each study: first author, year of publication, study population characteristics, study design, inclusion/exclusion criteria, intervention characteristics, assessment of the outcome, and results. For this review’s purpose, we had not extracted information regarding satisfaction and adherence to intervention (as the outcomes).

## 3. Results

### 3.1. Evaluation of the Study 

**Figure 1:**
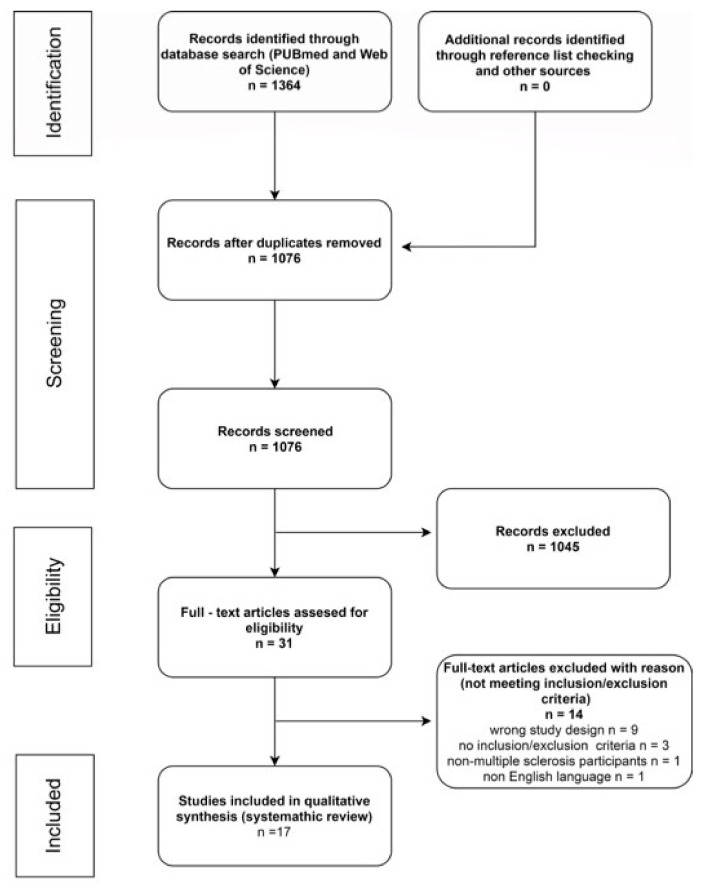
Study flow diagram.

From a total of 1364 only 17 studies were included. The search results and the flow diagram of the study selection are summarized in Figure 1.

Although the overall number of participants from all the studies was 904, two of the studies were conducted on the same group of participants [19,20]. Thus, a total of 854 participants were involved in the studies included. All participants were diagnosed with MS. In 10 studies [19,20,21,22,23,24,25,26,27,28], participants had to be diagnosed according to the McDonald criteria (2010 [29] or 2017 [30] revision—depending on the date of the study conduction).

Using QATQS, 11 studies were deemed to be ‘strong’, 5—‘moderate’ and 1—weak’. Detailed results are presented in Table 1.

**Figure 2:**
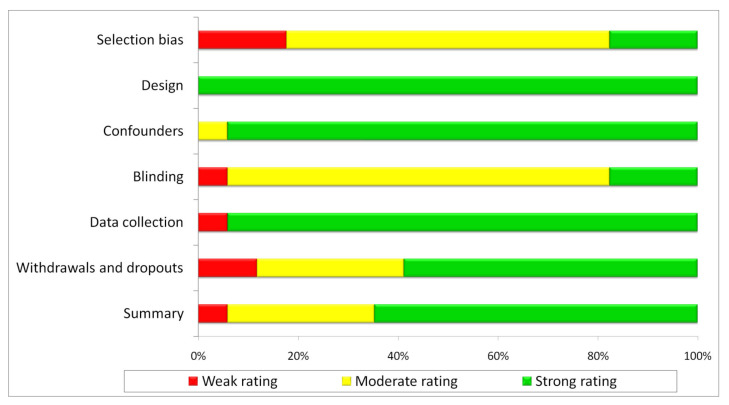
Review authors’ judgements about each quality section item presented as percentages across all included studies.

Study design, confounder control, and data collection methods sections were rated highest, while selection and blinding sections overall scored as weakest. The results for individual sections are presented in Figure 2.

### 3.2. Characteristics of Research Participants and Study Criteria

Participants’ characteristics are presented in Table 2, Table 3 and Table 4. The studies were selected according to a selected function in MS patients and divided into subgroups: studies of new technologies and balance and gait parameters; studies of new technologies and hand function; studies of new technologies and other health-related outcomes.

Of all the studies included: five [19,20,22,26,35] involved intervention participants conducted at home, six were conducted using Xbox Kinect [19,20,24,25,27,31], and seven studies reported no adverse outcomes [23,24,25,26,33,36,37].

### 3.3. The Impact of Applying of New Technologies on Balance and Gait Parameters

The influence of new-technology-based/assisted training on balance and gait parameters was the subject of the majority of the studies included [19,20,22,24,25,26,27,31,33,34,35,37]. 

Characteristics of the studies are presented in Table 5.

Conroy et al. [22] compared two physiotherapy training programs. In the intervention group (IG), there were exercises with asynchronous text messaging for exercise updates from the therapist via communication application, while there were only exercises prescribed at baseline in the control group (CG). The authors failed to observe any improvements in regard to the parameters measured (balance and gait) both in IG ( [Mean difference (*p*-value)] respectively: six-minute walk test [1.6 (0.28)]; the Berg Balance Scale (BBS)— [−4.6 (0.92)]; MS Walking Scale-12 [−2.7 (0.07)]; Timed 25-foot walk [0.0 (1.0)]) and in CG ( [Mean difference (*p*-value)] respectively: six-minute walk test [71.8 (0.38)]; BBS [2.3 (0.22)]; MS Walking Scale-12 [1.6 (0.76)]; Timed 25-foot walk [0.4 (0.38)]). However, the authors pointed out that the high attrition rate in their study (only 24 from 54 participants originally enrolled at the baseline completed the study), which resulted in a small sample size, may be the factor that reduced the possibility to detect changes in the population studied.

Maggio et al. [24] reported significant improvements in regard to balance and neuropsychological parameters in both experimental (receiving VR-based, semi-immersive motor and cognitive rehabilitation) and control (receiving only conservative rehabilitation) groups. However, an increase in all the parameters measured was observed only in the group receiving VR-based intervention. The authors concluded that VR can be an effective tool for functional recovery in MS patients.

Molhemi et al. [25] found that VR-based balance training can be as efficient in balance improvement and reduction of fall risk as conventional physical exercises. Those authors [25] reported that certain effects of VR-based training lasted longer than the effects of exercises. The authors observed an improvement in regard to the Timed Up and Go test, and the reaction time remained significant at three-month follow-up only in the group receiving VR-based training. 

Another study included in this review [26] proved that home-based balance rehabilitation, in the form of exercises on a balance platform with visual and auditory feedback, can be beneficial to MS patients. Novotna et al. [26] showed that the above-mentioned training conducted for four weeks improved balance among participants at the end of the study period (in terms of the BBS and Mini-BESTest *p* = 0.001) and after four weeks of follow-up (in terms of the BBS and Mini-BESTest *p* = 0.001). However, no changes regarding the gait parameters measured were observed. Those authors pointed that despite the total improvement in the BBS (mean 1.9 points) being lower than the minimal clinically important difference (3 points), home-based, individually adjusted balance training could improve balance in a clinically observable manner. In their opinion, the improvement was clinically insufficient (although statistically significant) due to the too-short time of intervention, which they pointed out as a study limitation [26].

Two research papers, authored by Ortiz-Gutierez et al. [19,20], studied the effectiveness of telerehabilitation on improving balance parameters. Both papers reported results of a study conducted on the same group of participants (which was confirmed via contact with the corresponding author of both papers). The results of the above-mentioned papers [19,20] showed greater, significant improvement in terms of the BBS (F = 29.896, *p* < 0.001), Tinetti Test (F = 46.898, *p* < 0.001) and Composite Equilibrium Score (part of The Sensory Organization Test) (F = 37.873, *p* < 0.001) in the group receiving telerehabilitation compared to the controls (conventional rehabilitation treatment). 

Peruzzi et al. [33] studied the effect of VR on treadmill training effectiveness regarding balance and gait among MS patients. The results showed significant improvements in both groups (treadmill training as control and treadmill training with VR as intervention group) in terms of gait speed, cadence, and stride length. Significantly more considerable improvements in the knee (*p* < 0.013) and hip (*p* < 0.001) range of motion were observed in the VR with treadmill group. The authors [33] also explained that the increase in lower limb joint kinematics, increased hip power generated at the terminal stance, and increased peak ankle power generated at push-off (which were more pronounced in the VR with treadmill group) were facilitated by the need to negotiate the virtual obstacles.

Robinson et al. [34] investigated the effects of exergaming on postural sway and gait in people with MS in comparison to traditional balance training and no intervention (control group). Improvements were present in the exergaming group compared to the control, although there were no statistically significant differences between the exergaming group and the group receiving traditional balance training. 

Lozano-Quilis et al. [31] studied the effectiveness of a Kinect-based system named RemoviEM, which is designed to motivate patients and give them visual feedback during motor rehabilitation. Comparing standard rehabilitation with rehabilitation using RemoviEM, these authors found differences in BBS improvement over time in favor of the group that used RemoviEM. In regard to other balance parameters measured, they found similar improvements in both groups. The authors emphasized that because most of the participants admitted that they had fun during the exercises, this type of using new technologies in rehabilitation may have a beneficial motivational effect.

Yazgan et al. [37] compared two exergaming treatments (with commercial exergames on Nintendo Wii Fit—group 1 and Balance Trainer device with games especially designed to train balance—group 2) with the control group (comprised of waitlist participants). The authors [37] found improvements in both intervention groups regarding the BBS; the mean difference in group 1 was 5.80 (SD = 5.29) and in group 2 2.66 (SD = 1.92). Improvements were significant in the comparison between both intervention groups and CG (*p* < 0.001), as well as in the comparison between group 1 and group 2 in favor of group 1 (*p* < 0.001). Statistically significant differences in favor of the intervention groups compared to the control were also observed regarding other outcomes measured (Timed up and go test, 6-min walking distance, Fatigue Severity Scale, and Multiple Sclerosis International Quality Of Life Questionnaire). The authors stated that exergaming, both with Nintendo Wii Fit and Balance Trainer, is fun, enjoyable, and competitive, which can improve exercise effectiveness [37]. 

The effect of treatment using new technologies on manual dexterity was studied in other four studies [23,27,28,36]. Cuesta-Gómez et al. [23] studied the effect of 10-week VR training of the hand (added to traditional rehabilitation of the hand) on manual dexterity. As an intervention, the authors [23] used a set of games prepared especially for this purpose and proved it to be successful at improving manual dexterity. The results of the Purdue Pegboard Test (more affected side (*p* = 0.032), both hands (*p* = 0.019), assembly (*p* = 0.008)) and the Box and Blocks Test (on the more affected side (*p* = 0.036)) improved significantly in the group which received VR training in comparison to CG (which was constituted by participants receiving only conventional rehabilitation treatment). The change in the above-mentioned parameters proved to be persistent after one-month follow-up in regard to the Box and Blocks Test on the more affected side (*p* = 0.010). The change between the groups in the Nine Hole Peg test score on the more affected side also proved to be significant (*p* = 0.011) in follow-up evaluation. Although this intervention was conducted at the study site, the authors pointed out that future studies of their training system should be conducted as an at-home rehabilitation system [23]. 

Upper limb function was also the topic of another study included in the present systematic review. Ozdogar et al. [27] found that the video-based exergaming performed for 8 weeks resulted in improvements in the Nine Hole Peg test score compared to baseline. The observed effect was significant both in the group treated with exergaming and in the group treated with a conventional rehabilitation program. No significant differences between the two treated groups were found. The authors [27] also demonstrated that differences between CG and both treated groups were statistically significant. An improvement in most cognitive function, leg function and balance-related outcome measures in the treated groups was also observed.

Pawlukowska et al. [28] conducted a study of the effect of computer-assisted hand therapy versus traditional hand therapy. The authors proved that adding computer-assisted exercises, oriented at improving attention, concentration, learning, and executive functions in standard hand therapy, is beneficial for MS patients. In the population studied, an improvement in time-to-complete The Nine Hole Peg Test in the computer-assisted therapy group was significant for dominant (*p* = 0.007) and for non-dominant (*p* = 0.037) hand, which was not observed in the control group [28]. 

Another study concerning manual dexterity was conducted by Walino-Peniagua et al. [36]. The authors failed to prove the effectiveness of game-based VR training in addition to occupational therapy in improving the function of the hand. In both intervention (VR+ OT) and control group (OT only), improvements were observed regarding the precision and effectiveness of specific functional tasks (such as picking small objects). However, the results did not differ significantly between both groups. The authors [36] hypothesized that the observed lack of differences may have been caused by the small sample size and high attrition rate in their study, which could possibly have compromised the results. 

Another study, by Norouzi et al. [32], investigated the effect of VR bimanual coordination training on bimanual coordination among persons diagnosed with MS. In the three-arm, randomized, controlled trial, the authors demonstrated improvement in all three groups (VR, VR+ conventional rehabilitation, and conventional rehabilitation only). However, the greatest impact on the bimanual coordination accuracy and consistency was observed in the group receiving combined intervention (VR+ conventional rehabilitation). The authors [32] emphasized that treatment comprised of both VR and conventional rehabilitation can be more beneficial to MS patients, and its effects can last longer. Table 6 presents characteristics of the studies.

Tallner et al. [35] investigated the effectiveness of an internet-based exercise intervention on improving health-related quality of life, muscle strength, respiratory function, physical activity, and fatigue among MS patients. The CG was comprised of waitlist participants. After three months from study baseline, CG participants received the same intervention as IG had received from the beginning. Thus, a between-group comparison was conducted based on the data obtained at assessment performed three months after the study beginning. The authors found that internet-based training intervention did not influence the health-related quality of life and fatigue, but muscle strength of the lower extremities, lung function and physical activity improved significantly. The results obtained after three months of training showed that the maximum muscle strength of the knee increased (by 9% and 13% in extensors and flexors of the knee, respectively). The authors indicated that the lack of direct supervision during training could be the factor potentially limiting the outcome and influencing the correctness of the performed exercise [35].

Charvet et al. [21] evaluated the effect of a home-performed, computer-based adaptive training program on MS participants’ cognitive functioning. This cognitive training was comprised of adaptive exercises aiming at improving speed, attention, working memory, and executive function through the visual and auditory domains. In the intervention group (IG), during the exercises, participants also received visual/ audio stimulation (initially, the auditory signals were slowed down to make them easier to remember; similarly, the visual signals were more contrasting than in the later stage of the exercises). The intervention group had a significantly higher change in the neuropsychological composite at the end of the study (estimated difference = 0.16 with 95% CI: 0.02 ± 0.30, *p* = 0.0286). The authors emphasized that their intervention can be successfully provided to MS patients at home. Characteristics of those studies are shown in Table 7.

In March 2020, the WHO declared the COVID-19 pandemic [38]. Since then, the virus has spread widely and rapidly. In the face of a lack of effective therapy against SARS-CoV 2, it is crucial to prevent infection. The preventive measures include increased hygiene and disinfection, social distancing, and avoiding unnecessary contact with other people [39,40]. For this reason, in order to provide care to vulnerable patients with an increased risk of developing COVID-19 and its severe course, healthcare requires reorganization and the use of new solutions.

In the situation of the COVID-19 pandemic, patients with neurological disorders may be deprived of their usual care [41]. During the pandemic, there was a tendency to implement home exercise programs due to the limited duration of therapy and access to physical therapy [42]. Home self-rehabilitation is an increasingly common element of rehabilitation programs, especially in the case of various long-term conditions such as MS. Patient adherence to physiotherapy programs recommended to be performed at home is crucial for the success and effectiveness of therapy and some studies show that patients who follow the prescribed program have better treatment outcomes [43,44]. Although adherence to prescribed home physiotherapy regimens is considered particularly important for a successful rehabilitation outcome, there are studies that show compliance problems between clinic and home self-exercise and non-adherence is often very high [45,46]. Participants are considered not to adhere to home rehabilitation regimens if they do not achieve the specified recommended repetition values [47], the recommended exercise duration [48], and the recommended frequency of exercise [49]. The reasons for non-compliance include the lack of support and supervision, no need to change lifestyle, lack of immediate relief of symptoms, and doubts and uncertainty about the therapy [50]. Previous research shows that adherence to recommendations for home rehabilitation is determined by the following factors: intention to engage in independent exercise, self-motivation, self-efficacy, prior exercise adherence [51], and social support [52]. Social support is believed to facilitate adherence by encouraging optimism and self-esteem, lowering stress related to illness, reducing depression, and providing practical help [52]. Understanding the factors influencing independent exercise at home gives researchers and practitioners greater opportunities to improve adherence by designing and implementing interventions aimed at reinforcing positive factors and minimizing barriers to compliance [52]. This also applies to the design of telerehabilitation using VR systems.

The current health system contingency due to the COVID-19 pandemic requires an acceleration in the use of telemedicine to enable neurorehabilitation outside the traditional settings such as hospitals, rehabilitation centers, private practices, and in the community. Teletherapy may replace and complement in-person treatment to mitigate constraints on service delivery that currently limit access to rehabilitation care.

The review shows that TR is applicable in the cognitive rehabilitation of patients with MS, which may also be particularly important in the COVID-19 pandemic situation. The review has demonstrated that in most studies rehabilitation interventions using new technologies have produced similar results to those obtained by direct exercise and sometimes even better results than those observed in traditional schemes. TR has proven to have a positive effect not only on general fitness but particularly on gait, balance, and upper limb function in MS individuals [19,20,23]. Tallner and co-authors [35] analyzed TR exercise programs in MS patients and observed that features promoting self-directed care and internet access to individualized tele-management resources were beneficial to physical activity and function.

Telemedicine, virtual reality, and gamification seem to be effective rehabilitation tools especially in the MS patient group. The systematic review presented confirms good tolerance and therapeutic effects in this group of patients. Additionally, most of the studies analyzed had not reported any harmful effects of the interventions. 

Moti with co-authors [53,54] showed that the content of web-based programs caused essential and remarkable increase in physical activity in research highlighting self-efficacy and use of a social cognitive-behavior-based framework. To be effective, both physical and cognitive rehabilitation programs should be intensive and sustained. Current limited access to cognitive rehabilitation can impair the relationship between the patient and medical staff. Remote communication technologies are increasingly seen as potential, effective options for supporting healthcare interventions including neurorehabilitation and cognitive rehabilitation [21]. The presented research shows a wide range of communication formats using the Internet, teleconferences, VR and a variety of interventions aimed at improving physical activity, cognitive function, education, and reducing fatigue. The promising effect of gamification in MS patients also manifests as its positive emotional impact. Gamification rewards users with numerical values. The scoring system associated with gaining rewards for achieving a certain number of points allows the patient to feel positively motivated and also influences their emotional skills such as self-satisfaction and self-esteem. Immediate feedback during gamification informs the patient about their current progress and the level of rehabilitation. This is ensured by auditory, visual and textual feedback that appears immediately after the patient’s action and informs them about their progress and the results of the exercises performed.

Numerous studies have proven in-home interventions to be effective, which is of great importance presently. Remote communication technologies seem to be measures of high effectiveness in rehabilitating and supporting MS patients, especially during the COVID-19 pandemic, as the traditional rehabilitation option is less accessible or in some cases inaccessible for patients. Therefore, it would be useful to confirm the effectiveness of this promising rehabilitation treatment option and conduct long-term research involving large samples including MS patients with severe and progressive disability. 

The present paper has some limitations. First, we have included only English-language articles from two databases in the analysis. The second limitation may be that despite the promising results of treatment involving new technologies in MS patients, only five of the studies assessed the effectiveness of home-based therapy. Therefore, in relation to the effectiveness of outpatient therapies involved in this review, possible discrepancies regarding therapy adherence may occur in the case of home treatment. This could possibly compromise the therapy effectiveness as compared to the studies analyzed.

## 4. Conclusions

The COVID-19 pandemic required a rapid adoption of new technologies to improve access to healthcare, as social distancing became mandatory. The present review proves telerehabilitation to be an effective motivational tool to restore and maintain both physical and cognitive function in patients with MS. In addition to improving motivation, another advantage of gamification is the possibility of choosing a convenient time of day for exercise, as well as reducing the costs associated with traveling to the clinic.

Further studies are needed to confirm the effectiveness of this promising rehabilitation treatment option. In particular, it would be useful to conduct long-term research involving large samples including MS patients with severe and progressive disability.

## Figures and Tables

**Table 1:** Evaluation of the methodological quality of each study using QATQS.

	Selection Bias	Design	Confounders	Blinding	Data Collection Methods	Withdrawals and Dropouts	Summary	
Chervet 2017 [21]	+	+	+	+	+	+	+	
Conroy 2017 [22]	?	+	+	?	+	-	?	
Cuesta-Gomez 2020 [23]	?	+	+	?	+	+	+	
Lozano-Quilis 2014 [31]	-	+	+	?	+	+	?	-
Maggio 2020 [24]	?	+	+	?	+	+	+	**Weak**
Molhemi 2020 [25]	?	+	+	?	+	+	+	
Norouzi 2020 [32]	+	+	+	?	+	+	+	
Novotna 2019 [26]	?	+	?	?	+	+	+	
Ortiz-Gutierrez 2013 (a) [19]	?	+	+	?	+	+	+	?
Ortiz-Gutierrez 2013 (b) [20]	?	+	+	?	+	+	+	**Moderate**
Ozdogar 2020 [27]	?	+	+	?	+	+	+	
Pawlukowska 2020 [28]	-	+	+	+	+	-	-	
Peruzzi 2017 [33]	?	+	+	?	+	?	+	+
Robinson 2015 [34]	?	+	+	-	+	?	?	**Strong**
Tallner 2016 [35]	+	+	+	?	-	?	?	
Waliño-Peniagua 2019 [36]	-	+	+	+	+	?	?	
Yazgan 2020 [37]	?	+	+	?	+	?	+	

**Table 2:** Characteristics of research participants in studies of new technologies and balance and gait parameters in MS patient groups.

Study (Author and Year)	Number of Participants	Age (Years as Mean (SD))	Inclusion Criteria	Exclusion Criteria
Conroy 2017 [22]	N = 51 (24 completed the study), IG = 26(16 completed the study); CG = 25 (8 completed the study)	All = 51 (8.1) IG = 50.4 (8.1) CG = 54.3 (5.9)	Age 18–65 years, confirmed MS diagnosis using McDonald MS diagnostic criteria, The Patient Determined Disease Steps score range of 2–6; and ability to use the “MS HAT” platform with modifications as needed; 25fW ≤ 3 min; ability to perform exercise independently or have an identified caregiver provided assistance; access to a working telephone line	MS exacerbation within 3 months of enrolment, a corticosteroid course within 60 days of screening, any medical condition or cognitive impairment that would interfere with exercise performance or understanding
Maggio 2020 [24]	N = 60; IG = 30; CG = 30	All = 50.0 (11.4) IG = 51.9 (9.9) CG = 48.2 (12.2)	MS diagnosis according to the McDonald criteria, stable in therapy for at least 6 months before the study entry; mild/moderate cognitive impairment (Montreal Cognitive Assessment > 18); absence of severe medical and psychiatric illness potentially interfering with the VR training; absence of disabling sensory alterations	Age >75 or <18 year; severe medical and psychiatric illness according to the Diagnostic and Statistical Manual of Mental Disorders, 5th Edition and International Classification of Disease; MS clinical and/or neuroradiological relapse in the 6 months before enrolment; EDSS > 7
Molhemi 2020 [25]	N = 39 (35 completed the study); IG = 19 (17 completed the study) CG = 20 (18 completed the study)	All = n/d IG = 36.8 (8.4) CG = 41.6 (8.4)	Confirmed diagnosis of relapsing-remitting or secondary progressive MS according to the McDonald criteria by a neurologist specialized in treating MS, aged 18–64 years, EDSS < 6, and BBS < 53	Exacerbation of symptoms in the past 3 months, MMSE < 24, neurologic or musculoskeletal diagnosis except MS that negatively affected their gait and balance, uncorrected visual or auditory impairments, pregnancy
Nowotna 2019 [26]	N = 39; IG = 23; CG = 16	All = 40.69 (10.2) IG = 39.39 (9.68) CG = 42.56 (10.63)	Clinically stable MS, without relapse or worsening in the previous three months; aged 18–60 years; ability to walk with or without a walking aid for at least 5 m; and ability to maintain a standing position for at least 10 min, ability to perform exercise (assessed by physiotherapist)	Inpatient rehabilitation program during the previous 3 months; orthopedic problems or other conditions affecting balance and gait performance; blurred vision; severe cognitive impairment or psychiatric disorders; pregnancy; weight over 150 kg
Ortiz-Gutierrez 2013(a) [19]	N = 50 (47 completed the study); IG = 25 (24 completed the study); CG = 25 (23 completed the study)	All = n/d IG = 39.69 (8.13) CG = 42.78 (7.38)	Age between 20 and 60 years; confirmed diagnosis of MS for over 2 years based on the McDonald criteria; medically stable during the 6 months prior to baseline; impaired balance with demyelinated lesions in the cerebellum and its connections demonstrated by Magnetic Resonance Imaging; EDSS score ranging from 3 to 5; Hauser ambulatory index value > 4; MMSE ≥ 24; no visual deficits; internet connection at home	Diagnosed with another disease or pathological condition that affects balance; had a relapse in the month prior to baseline or during the intervention process, received steroid cycle prior to beginning the evaluation protocol and within the 4 months duration of the project intervention
Ortiz-Gutiérrez 2013 (b) [20]	N = 50 (47 completed the study); IG = 25 (24 completed the study); CG = 25 (23 completed the study)	All = n/d IG = 39.69 (8.13) CG = 42.78 (7.38)	Age between 20 and 60 years; confirmed diagnosis of MS for over 2 years based on the McDonald criteria; medically stable during the 6 months prior to baseline; impaired balance associated with demyelinated lesions in the cerebellum and its connections; EDSS from 3 to 5; Hauser ambulatory index value > 4, absence of cognitive impairment according to the MMSE, no visual deficits, internet connection at home	Diagnosis of another disease or pathological condition that affects balance; an attack in the month prior to baseline or during the intervention process; receiving a cycle of steroids 6 months prior to beginning the protocol and within the 4 months duration of the project intervention
Peruzzi 2017 [33]	N = 31 (25 completed the study); IG = 16 (14 completed the study); CG = 15 (11 completed the study)	All = n/d IG = 43.6 (10.2) CG = 42.0 (12.0)	Diagnosis of relapsing-remitting MS according to the McDonald criteria, an expanded disability status scale between 3 and 5.5 and, a MMSE ≥ 26, no relapses within the six months prior to the study	Chronic medical illnesses, severe visual deficits, severe ataxia or severe depression, botulinum toxin inj. within the past 4 months or functional surgery in the past 6 months.
Robinson 2015 [34]	N = 56; IG1 = 20, IG2 = 18(15 completed the study), CG = 18 (11 completed the study)	All = 52 (5.8) IG1 = 52.6 (6.1) IG2 = 53.9 (6.5) CG = 51.9 (4.7)	Male or female, aged 18–65 years, clinical diagnosis of MS, self-reported ability to walk 100 m with or without resting with the use of one stick or crutch (EDSS score of 6), able to read and comprehend written and spoken English	Acute exacerbation and/or relapse of MS symptoms within the last three months, diagnoses of any other condition affecting CNS, any musculoskeletal injury, or receiving physical therapy
Lozano-Quilis 2014 [31]	N = 11; IG = 6; CG = 5	All = n/d IG = 48.33 (10.82) CG = 40.60 (9.24)	Age 18–65 years, relapsing-remitting and secondary progressive MS, minimum score of 6 on all items of the domain of the Functional Independence Measure, do not need assistive devices for ambulation or at most a cane, do not have cognitive impairments	Flare-up symptoms, cannot physically complete all rehabilitation sessions
Yazgan 2020 [37]	N = 47 (42 completed the study); IG1 = 16 (15 completed the study); IG2 = 16 (12 completed the study); CG = 15	All = n/d IG1 = 47.46 (10.53) IG2 = 43.08 (8.74) CG = 40.66 (8.82)	MS patients followed up regularly at the MS Outpatient Clinic of our Neurology Department; ambulatory and volunteered to participate in the study; stable phase of the disease, without relapses or worsening in the last 3 months; EDSS between 2.5 and 6; age between 25 and 60 years	Diagnosis of any other disorder affecting CNS, musculoskeletal disorder, pregnancy, blurred vision, psychiatric problems or severe cognitive impairment

MS—multiple sclerosis, VR—virtual reality, BBS—the Berg Balance Scale, EDSS—Expanded Disability Status Scale, 25fW—The 25 foot walk test, NHPT—Nine Hole Peg Test, MMSE—Mini-Mental State Examination; CNS—the central nervous system. Age is expressed as: mean (standard deviation); IG—intervention group; CG—control group.

**Table 3:** Characteristics of research participants in studies of new technologies and hand function in MS patient groups.

Study (Author and Year)	Number of Participants	Age (Years as Mean(SD))	Inclusion Criteria	Exclusion Criteria
Cuesta-Gomez 2020 [23]	N = 30; IG = 16; CG = 14	All = 46.66 (2.04) IG = 49.86 (2.46) CG = 42.66 (3.14)	Diagnosis of MS according to the McDonald criteria with over 2 years evolution; a score of between 3.5 and 7.5 on the EDSS; with stable medical treatment during at least the 6 months prior to the intervention; muscle tone in the upper limbs ≤2 on the modified Ashworth Scale; ≤4 in the “Pyramidal Function” section of the EDSS functional scale; absence of cognitive decline (≥24 in the MMSE; and ≤2 in the “Mental Functions” section of the EDSS)	Diagnosis of another neurological illness or musculoskeletal disorder different to MS; the diagnosis of a cardiovascular, respiratory, or metabolic illness or other conditions which may interfere with the study; suffering a flare-up or hospitalization in the last 3 months. prior to commencement of the assessment protocol or during the process of the therapeutic intervention; receiving a cycle of steroids 6 months. prior to the commencement of the assessment protocol and within the study period of intervention; receiving treatment with botulinum toxin in the 6 months. prior to the beginning of the study; visual disorders non-corrected by optical devices
Ozdogar 2020 [27]	N = 60 (57 completed the study); IG1 (video-based exergaming group) = 21 (20 completed the study); IG2 (conventional rehabilitation) = 19 (17 completed the study); CG = 20	All = 40.1 (10.7) IG1 = 39.2 (8.6) IG2 = 43.6 (10.5) CG = 37.9 (12.4)	Relapsing-remitting or secondary progressive type of MS, being able to walk at least 100 m without resting, being able to stably stand for half an hour, relapse-free period of 3 months, willing to participate in the study	Another neurological disorder, relapse during the study period, orthopedic surgery history covering the ankle-foot, knee, hip, or spine, affecting balance, and diagnosis of severe cognitive and/or psychiatric impairment
Pawlukowska 2020 [28]	N = 40 (30 completed the study) IG = 20 (10 completed the study); CG = 20	All = n/d IG = 53.9 (n/d) CG = 49.6 (n/d)	Between 18 and 65 years, MS clinically diagnosed based on the McDonald criteria, EDSS range from 1.5–4 points, impairment of the upper limb, NHPT score <2 standard deviations (SDs) from the norm for their age and sex	MMSE score <26, alcoholism, and severe vision disorders including diplopia and coinciding upper limb therapy
Waliño-Paniagua 2019 [36]	N = 16; IG = 8; CG = 8	All = 46.44 (9.09) SG = 46.13 (9.49) CG = 46.75 (9.31)	A diagnosis of MS according to the McDonald criteria with over two years evolution; a score of between 3.5 and 6 on the EDSS (as well as a score ≤ 4 in the “Pyramidal Function” section of the EDSS functional scale, or score ≤ 2 in the “Mental Functions” section of the EDSS); stable medical treatment during at least the six months prior to the intervention; muscle tone in the upper limbs not greater than two points on the modified Ashworth Scale; absence of cognitive decline; ability to understand instructions and a score ≥ 24 in MMSE	Diagnosis of another neurological illness or musculoskeletal disorder different to MS; the diagnosis of a cardiovascular, respiratory, or metabolic illness or other conditions which may interfere with the study; suffering a flare-up or hospitalization in the last 3 months. prior to commencement of the assessment protocol or during the process of the therapeutic intervention; receiving a cycle of steroids 6 months. prior to the commencement of the assessment protocol and within the study period of intervention; receiving treatment with botulinum toxin in 6 mth. prior to the beginning of the study; presence of visual disorders non-corrected by optical devices
Norouzi 2020 [32]	N = 45; IG1 = 15 (VR); IG2(VR+ Conventional Physical Training) = 15; CG (Conventional Physical Training) = 15	All = 26.39 (3.45) IG1 = n/d IG2 = n/d CG = n/d	Female with MS; age 20–30 years; right handed; a diagnosis of poor fine manual dexterity (according to the NHPT criteria); signed written informed consent; normal vision based on the Snellen Chart Test; self-reported normal audition	Psychiatric issues (ascertained by a brief psychiatric interview—Mini International Neuropsychiatric Interview); intake of mood- and arousal-medications or substances; orthopedic problems; pregnancy; and somatic diseases such as diabetes

MS—multiple sclerosis, VR—virtual reality, BBS—the Berg Balance Scale, EDSS- Expanded Disability Status Scale, 25fW—The 25 foot walk test, NHPT—Nine Hole Peg Test, MMSE—Mini-Mental State Examination. Age is expressed as: mean (standard deviation); IG—intervention group; CG—control group.

**Table 4:** Characteristics of research participants in studies of new technologies and other health- related outcomes in MS patients.

Study (Author and Year)	Number of Participants	Age (Years as Mean(SD))	Inclusion Criteria	Exclusion Criteria
Tallner 2016 [35]	N = 126 (78 completed the study); IG = 59 (36 completed the study), CG = 67 (41 completed the study)	All = 40.8 (9.9) IG = 40.9 (10.4) CG = 40.7 (9.5)	Diagnosed multiple sclerosis, an EDSS ≤ 4.0, not less than four weeks of clinical stability prior to inclusion in the study, access to the Internet	Primary progressive multiple sclerosis and clinically relevant cardiological, internal, or orthopedic contraindications to exercise, which were assessed by the patients’ attending physicians
Charvet 2017 [21]	N = 135; IG = 74; CG = 61	All = 50 (12) IG = 52 (11) CG = 48 (13)	Meeting diagnostic criteria for MS (McDonald criteria), scoring one or more standard deviations below published normative data on the Symbol Digit Modalities Test; reading recognition standard score of 85 or above (Wide Range Cognitive Achievement Test Third Edition); learned English by age 12 years; adequate visual, auditory, and motor capacity to operate computer software; no anticipated medication changes during the course of the three-month study period, and no relapses or steroids in the previous month	History of any developmental disorders, conditions other than MS associated with cognitive impairment, a primary psychiatric disorder, any serious medical conditions, alcohol or substance use disorder, history of use of computer-based CT developed by Posit Science (the developer of the study program)

MS—multiple sclerosis, VR—virtual reality, BBS—the Berg Balance Scale, EDSS—Expanded Disability Status Scale, 25fW—The 25 foot walk test, NHPT—Nine Hole Peg Test, MMSE—Mini-Mental State Examination. Age is expressed as: mean (standard deviation); IG—intervention group; CG—control group.

**Table 5:** Characteristics of studies analyzing the effect of new technologies on gait and balance in MS patients.

Study (First Author and Year)	Study Design	Type of Therapeutic Intervention	Intervention Description	Frequency and Duration of Sessions	Period of Therapeutic Intervention (Number of Sessions)	Measured Domains	Measurements	Key Results
Conroy 2017 [22]	single-blinded, randomized controlled trial	Internet-supported exercise	CG—individualized exercise prescriptions in paper hand-out form common for physiotherapy home exercise programs. IG—the baseline written exercises and access to asynchronous text messaging for exercise updates from the therapist via the “MS HAT” platform. No live on-line exercise supervision	n/d	6 months (n/d)	Balance, gait	25fW, 6MW, BBS, MSWS12	No improvements in regard to walking ability and balance in IG and CG
Maggio 2020 [24]	single-blinded, randomized controlled trial	VR-based, semi-immersive motor and cognitive rehabilitation	All participants underwent a standard physical treatment consisting of general conditioning exercises and cognitive rehabilitation. IG—cognitive training was performed using VR, CG—conventional cognitive training	Cognitive training 60 min 3 x/wk. General conditioning training 30 min—no data regarding frequency	8 wks (24)	The neuropsychological battery test markers, i.e.,: depression, recall, quality of life, balance	Montreal Cognitive Assessment; BDI; Rey-Osterrieth complex figure test; Multiple Sclerosis Quality of Life-54; Paced auditory serial addition task for two seconds; Spatial recall test; TUG; Tinetti scale	Improvements in Tinetti scale, Rey–Osterrieth complex figure test; Multiple Sclerosis Quality of Life-54 and BDI were observed in both groups. Significant increase in visual perception, visuospatial abilities, short term visual memory, working memory and executive functions, speed of information processing, sustained attention and TUG test score was observed only in IG
Molhemi 2020 [25]	prospective randomized controlled trial	VR-based balance training	Participants in both groups received exercises including standing, walking, and weight-shifting. CG—standing exercise included multidirectional stepping, single and double-leg standing; walking exercise involved forward, backward, and side walking and weight-shifting, half-squat, leaning, and reaching. In the IG, progressive balance exercises were employed using the Xbox360 with Microsoft’s Kinect^®^ with “Light Race”, “Stack’em up”, and “20,000 leaks” exergames	35 min (5 min—warm-up; 30 min exercises) 3 x/wk	6 wks (18)	Balance in static and dynamic conditions	Single- and dual-task TUG, single- and dual-task 10 MWT, Dual Task Costs, BBS, MSWS12, Fall Efficacy Scale-international, Activities-specific Balance Confidence scale	At the follow-up, reaction time and the number of falls demonstrated significant differences favoring IG. At the follow-up, there were no significant between-group differences in regard to TUG, BBS, MSWS12, Fall Efficacy Scale-international, Activities-specific Balance Confidence scale
Novotna 2019 [26]	wait list randomized controlled study	Balance training with audio-visual biofeedback	IG—individually tailored home-based balance exercise training using Homebalance^®^ (therapeutic games where the therapeutic task can be set to different positions/directions, or the therapeutic task was to increase the limits of stability combined with cognitive training). CG—waiting list (no intervention)	At least 15 min x7/wk	4 weeks (28 (approx. 7 hrs in total))	Balance, gait parameters, falls	BBS; Mini-BESTest; TUG; assessment of the spatio-temporal gait parameters (by GAITRite walkway system). Falls Efficacy Scale, Activities-specific Balance Confidence Scale, MSWS12.	Statistically significant improvement in the mean BBS and in the Mini-BESTest. No improvement among other outcomes measured
Ortiz-Gutierrez 2013 (a) [19]	Non-blinded, non-randomized controlled trial	VR video games training	The CG—physiotherapy treatment (low-loads strength exercises, proprioception exercises on unstable surfaces and gait facilitation exercises, and muscle-tendon stretching). IG—individual TR treatments using the Xbox360^®^ console with Microsoft^®^ Kinect. The protocol proposed tasks such as throwing and hitting objects with one’s hands and feet, hitting and receiving balls with different body parts, dodging objects, overcoming obstacles, imitating postures, or managing virtual elements	SG 20 min 4 x/wk; CG 40 min x2/wk	10 wks (SG—40 (up to 800 min)in total, CG—20 (up to 800 min) in total)	Posturography parameters, balance	BBS; Tinetti scale; Computerized Dynamic Posturography (The Sensory Organization Test and the Motor Control Test)	BBS and Tinetti scale scores revealed significant between-group differences in the IG, achieving higher values. Composite Equilibrium Score (part of The Sensory Organization Test) was significantly higher in IG in comparison with CG at the post intervention assessment
Ortiz-Gutiérrez 2013 (b) [20]	Non-blinded, controlled trial	VR-based balance training	IG received individual treatments using the Xbox 360 TM console with MicrosoftTM Kinect following a protocol consisting of three games (Kinect SportsTM, Kinect Joy RideTM, and Kinect AdventuresTM). CG received physiotherapy treatment based on low-loads strength exercises, proprioception exercises on unstable surfaces and gait facilitation exercises, muscle-tendon stretching	SG 20 min 4 x/wk; CG 40 min x2/wk	10 weeks (SG—40 (up to 800 min) in total, CG—20 (up to 800 min) in total)	Posturography parameters, postural control	Computerized dynamic posturography (by Smart EquitestTM Version 8.2 CDP device); The Sensory Organization Test	Statistically significant improvement in composite equilibrium score in IG, non-significant improvements in CG
Peruzzi 2017 [33]	single-blinded, randomized controlled trial	VR-based treadmill training	A medical treadmill with a harness was used to administer the training programs in both groups. The IG—were walking on the treadmill while watching a virtual tree-lined trail (passing the obstacles appearing on the trail and following a road map, which was shown to them at the beginning of each walking bout). CG -only treadmill training with no VR	45 min 3 x/wk	6 wks (18)	Gait parameters, walking endurance and speed, mobility, balance, obstacle negotiation, disability	Gait analysis (gait data were collected using a six-camera stereophotogrammetric system with two force platforms, gait analysis was carried using the Motion Capture software (Vicon Nexus 2.0, Plug-in Gait), 6 MWT,10 MWT, TUG, BBS, four square step test, timed test consisting of stepping over an obstacle, Expanded Disability Status Scale)	Both the IG and CG significantly improved gait speed, cadence and stride length. Significantly larger improvements in kinematics and kinetics of gait in IG (knee range of motion *p* < 0.013, hip range of motion *p* < 0.001)
Robinson 2015 [34]	prospective, randomized controlled three-arm trial	Video-based exergaming balance training	IG 1 received exergaming with Wii Fit™; IG2 received traditional balance training, and CG received no intervention	40–60 min 2 x/wk	4 wks (8)	Postural sway, gait, walking ability, perceived activity and participation restrictions	Force plate, GAITRite™ walkway, MSWS12, 12-item World Health Organization Disability Assessment Schedule 2.0 questionnaire	Greater improvement in balance scores in all three of measures of postural sway in the IG1 group when compared to CG, and in postural sway antero-posterior and medio-lateral range in IG2 when compared to the CG group. No significant differences were found between IG1 and IG2 in outcome measured
Lozano-Quilis 2014 [31]	single-blinded, randomized controlled trial	VR-based balance training	In each session, CG—standard balance and gait rehabilitation exercises. IG—45 min performing the same (as in CG) exercises, and 15 min of the virtual rehabilitation exercises	60 min 1 x/week; SG—45 min of exercises and 15 min of VR; CG—60 min of exercises	10 weeks (10)	Balance in static and dynamic conditions	TBB; Tinetti; the Single Leg Balance test; 10 MWT; TUG	Significant group-by-time interaction was detected in the scores for the BBS (*p* = 0.030) and Single Leg Balance test of right foot (*p* = 0.033)
Yazgan 2020 [37]	single-blinded, randomized controlled, three-arm trial	Exergaming program	IG1—exergaming program (Nintendo Wii Fit) based on exergames selected from the Wii Fit Plus balance games section. IG2—exergaming program (Balance Trainer) CG participants were placed on a waiting list and invited to start exercising using Nintendo Wii Fit or Balance Trainer after the end of the study period	60 min 2 x/wk	8 wk (16)	Balance, gait, mobility, fatigue, quality of life	BBS, TUG, 6 MWT, Fatigue Severity Scale, Multiple Sclerosis International QoL Questionnaire	Statistically significant improvement in IG1 and IG2. IG1 noted better improvements than CG in balance tests, walking efficiency, fatigue and quality of life. IG2 was superior to CG in regard to balance, fatigue and QoL improvements

MS—multiple sclerosis, VR—virtual reality, BBS—the Berg Balance Scale, EDSS—Expanded Disability Status Scale, 25fW—The 25 foot walk test, QoL—quality of life, IG—intervention group; CG—control group.3.4. The Impact of Applying New Technologies on Hand Function

**Table 6:** Characteristics of studies analyzing the effect of new technologies on hand function in MS patients.

Study (First Author and Year)	Study Design	Type of Therapeutic Intervention	Intervention Description	Frequency and Duration of Sessions	Period of Therapeutic Intervention (Number of Sessions)	Measured Domains	Measurements	Key Results
Cuesta-Gomez 2020 [23]	single-blinded, randomized controlled trial	Leap Motion Controller (LMC) System	CG—a specific upper limb conventional motor rehabilitation therapy (60 min) (joints mobilization, muscles strengthening, functional task practice). IG—the same conventional motor rehabilitation therapy (45 min) plus VR (Leap Motion Controller) (15 min). Six serious games were performed first unilaterally and then bilaterally	60 min 2 x/wk	10 wks (20)	Grip Strength; gross manual dexterity on both sides; speed and motor dexterity of each hand; handfunction; fatigue; physical and psychological well-being	Grip strength (dynamometry); The Box and Blocks Test; TPPT; NHPT; Fatigue Severity Scale; Multiple Sclerosis Impact Scale	Significant improvements in IG in comparison with CG were found for the TPPT on the more affected side, both hands, assembly, and the Box and Blocks Test on the more affected side. For the follow-up measurements, significant improvements were found for The Box and Blocks Test on the more affected side and the NHPT on the more affected side
Ozdogar 2020 [27]	three-arm, non-blinded randomized controlled trial	Video-based exergaming therapy of arm and cognitive function	The video-based exergaming (The Kinect Sports Rivals game) was applied in IG. In CG patient-specific rehabilitation program (included balance, arm, and core stability exercises) was applied to the participants	45 min 1 x/wk	8 wks (8)	Hand dexterity, unilateral and bilateral activities of daily living, cognitive functioning, lower limb and trunk strength and endurance, gait, balance, depression, fatigue, QoL	NHPT; The Manual Ability Measurement-36; The Brief International Cognitive Assessment in MS; The Activities-specific Balance Confidence; Sit-to-stand test; The curl-up; 25 fW; MSWS12; The Six Spot Step Test; BDI; The Modified Fatigue Impact Scale; The Multiple Sclerosis International QoL	Significant improvements in the arm functions and in the most cognitive function, leg function and balance-related outcome measures were observed in the IG1 and IG2. No significant difference was observed in the changes from baseline at 8 weeks in the study outcomes between the IG1 and IG2 while several significant differences were observed in the changes of the CG compared to the IG1 and IG2
Pawlukowska 2020 [28]	double-blinded, randomized controlled trial	Computer-assisted hand therapy	Participants in each group received progressive hand therapy treatments. IG—upper limb treatment with the RehaCom cognitive function platform (object moving along a pre-defined track and a cursor controlled by a joystick). CG—only progressive hand therapy treatments without RehaCom hand therapy	SG 20 min 3 x/wk; CG = n/d	SG up to 3 mth; CG n/d IG (540–640 min. in total mean—586 min.); (CG n/d)	Hand dexterity	NHPT	Improvement in time-to-complete NHPT in IG in regard to dominant (*p* = 0.007) and non-dominant hand (*p* = 0.037). No significant improvements in CG
Waliño-Paniagua 2019 [36]	single-blinded, randomized controlled trial	Hand dexterity—Game-Based VR Video Capture Training Program	Both groups received conventional occupational therapy treatment. IG additionally received VR treatment sessions via the online and free website motiongamingconsole.com, during which they performed exercises with video capture of the upper limb movements	SG = 30 min of occupational therapy 2 x/week + 20 min of VR treatment 2 x/week; CG 30 min of occupational therapy 2 x/week	10 weeks (SG = 40 (20 sessions of occupational therapy + 20 sessions of VR therapy in total); CG = 20 (only occupational therapy))	Manual dexterity and coordination, hand functional capacity	TPPT, Jebsen-Taylor Hand Function Test, Grooved Pegboard Test	No significant differences between outcome measures among IG and CG. Statistically significant differences were found in picking up small common objects in both groups
Norouzi 2020 [32]	three-arm controlled study	VR bimanual coordination training	In IG1 (VR group)—coordination of the movement of both hands with the movements of a visual stimulus (Kinect captured the hand movements of the participants). CG (Conventional Physical Training)—performing a complete cycle of in–out-in handle displacements in time with the beat of metronome. The metronome begun to pace at slow frequency (58 bpm) for 20 s. The same coordination task was paced at a medium metronome frequency and at a fast metronome frequency (152 bpm). IG2 (VR + Conventional Physical Training)—combined therapy	30 min 2 x/wk	8 wks (16)	Bimanual coordination	Bimanual coordination assessing procedure: Participants received a general orientation to the task. The task required them to grasp two handles attached to the moving slides and displace them horizontally in the left-right dimension (wrist extension and flexion). While grasping the two handles, participants produced a 180° relative phase (anti-phase) pattern. Potentiometers encoded the displacement of the handles over a 20 s trial	Bimanual coordination accuracy and consistency improved over time from baseline to study completion and to follow-up, butmore so in IG2 than CG or IG1. Improvements were greater in IG1 compared to CG

MS—multiple sclerosis, VR—virtual reality, BBS—the Berg Balance Scale, EDSS—Expanded Disability Status Scale, 25fW—The 25 foot walk test, QoL—quality of life, IG—intervention group; CG—control group.3.5. The Impact of Applying New Technologies on Other Health-Related Outcomes in MS Patients

**Table 7:** Characteristics of studies analyzing the effect of new technologies and other health-related outcomes in MS patients.

Study (First Author and Year)	Study Design	Type of Therapeutic Intervention	Intervention Description	Frequency and Duration of Sessions	Period of Therapeutic Intervention (Number of Sessions)	Measured Domains	Measurements	Key Results
Tallner 2016 [35]	randomized, controlled trial with a wait list control group	Internet-Supported Physical Exercise Training	In IG the aerobic and endurance exercise training was home-based and supervised via the Internet. CG participants were instructed to maintain their previous physical activity behavior. After waiting three months, they received the same e-training intervention as the intervention group had received from the start	2 x/wk. of aerobic training and 1 x/wk. endurance training	6 months. in SG; 3 months. waiting and 3 months. of exercises in CG	Health-related QoL, fatigue, maximum isometric muscle strength of lower limbs, lung function, habitual physical activity	Hamburg QoL Questionnaire for MS, Würzburg Fatigue Scale for MS, M3 Diagnos machine (for lower limb strength test), Forced vital capacity and peak expiratory flow (by Master Screen CPX System), Baecke Questionnaire (German version)	No improvement in health-related QoL and fatigue, IG recorded significant increases in strength of the lower extremities, lung function (peak expiratory flow) and physical activity. Improvement was significant in comparison to CG in regard to muscle strength and physical activity
Charvet 2017 [21]	double-blinded, randomized, active-placebo-controlled trial	Computer-based adaptive cognitive training program	IG—online adaptive cognitive training program (with a set of 15 exercises targeting speed, attention, working memory, and executive function through the visual and auditory domains). CG—an intervention based on a software gaming suite developed by Hoyle Puzzle and Board Games (2008 version)	60 min 5 x/wk.	12 wks. (60)	MS- related cognitive impairment, change in cognitive functioning	Neuropsychological Composite Score, Self-Reported Change in Cognitive Functioning (participants rated whether their cognition stayed the same (0), improved (1) or declined (−1) from baseline to study end)	IG had a significantly higher change in the neuropsychological composite from baseline to study end than CG

MS—multiple sclerosis, VR—virtual reality, BBS—the Berg Balance Scale, EDSS—Expanded Disability Status Scale, 25fW—The 25 foot walk test, QoL—quality of life, IG—intervention group; CG—control group.4. Discussion

## Data Availability

The datasets generated for this study are available on request to the corresponding author.
